# Trends of Azole-Resistant *Aspergillus* Fumigatus Susceptibility Over 12 Years from a German ECMM Excellence Center

**DOI:** 10.1007/s11046-025-00941-x

**Published:** 2025-04-05

**Authors:** Hedda Luise Verhasselt, Lara Thissen, Ulrike Scharmann, Silke Dittmer, Peter-Michael Rath, Joerg Steinmann, Lisa Kirchhoff

**Affiliations:** 1 Institute of Medical Microbiology, Excellence Center for Medical Mycology (ECMM), University Hospital Essen, University of Duisburg-Essen, Virchowstraße 179, 45147 Essen, Germany; 2 Institute of Clinical Microbiology, Infectious Diseases and Infection Control, Klinikum Nürnberg, Paracelsus Medical University, Nuremberg, Germany

**Keywords:** *Aspergillus*, Azole-resistance, Manogepix, Olorofim, *CYP51A*

## Abstract

Numbers of infections with azole-resistant *Aspergillus fumigatus* (ARAf) were rising in the last decades. We assessed ARAf susceptibility trends towards five antifungal agents (amphotericin B (AMB), itraconazole (ITR), voriconazole (VCZ), olorofim (OLO) and manogepix (MGX)) over twelve years in a German Excellence Center for Medical Mycology (ECMM). In addition, underlying mutations were studied and correlated with trends in minimum inhibitory concentration (MIC). Broth microdilution (BMD) was performed following EUCAST guidelines for 143 clinical ARAf isolates collected between the years 2011 and 2022 in a West German tertiary care centre. BMD was carried out for all antifungal agents in the following concentration ranges: 0.016–8 mg/L for AMB, ITR and VCZ as well as 0.001–0.5 mg/L for OLO and 0.004–2 mg/L for MGX. Molecular assays on mutations associated with antifungal resistance were performed for all 143 isolates (AsperGenius® 1.0, Pathonostics, Maastricht, The Netherlands) and for a total of ten non TR_34_/L98H and TR_46_/Y121F/T289A mutated ARAf isolates additional *cyp51A* sequencing was carried out. For all isolates, microdilution revealed a MIC_50_ of > 8 mg/L for ITR, 4 mg/L for VCZ, 0.03 mg/L for OLO, 0.016 mg/L for MGX, and 0.5 mg/L for AMB. Considering EUCAST breakpoints, 97.9% of the strains (n = 140) were resistant to VCZ, 1.4% (n = 2) towards AMB and 92.3% towards ITR (n = 132). Molecular assays revealed 123 (86%) isolates with the azole resistance underlying mutation TR_34_/L98H, 10 (7%) with a TR_46_/Y121F/T289A mutation and 10 (7%) with other *cyp51A* mutations. A comparison of triazole MICs of isolates collected from 2011 to 2019 with the MICs of isolates collected between 2020 and 2022 revealed no significant differences for itraconazole (*p* = 0.543) and for voriconazole (*p* = 0.148),with a trend of increased geometric mean for ITR and VCZ MICs over time. MICs for OLO and MGX did not significantly differ between isolates with the distinct azole-resistance underlying mutations. Before 2016, the azole resistance underlying mutations were mainly TR_34_/L98H, but the portion of isolates with TR_46_/Y121F/T289A and other *Cyp51A* mutated isolates increased afterwards. We showed almost stable MICs for ITR and VCZ over twelve years in ARAf isolates from West Germany while occurring azole resistance underlying mutations varied with an increase in the proportion of TR_46_/Y121F/T289A and other *Cyp51A* mutations after 2016.

## Introduction

Mould infections are a public health concern posing globally crucial disease burden with considerable mortality rates ranging from 30 to 80% [1, 2]. *Aspergillus fumigatus* is the most common cause of aspergillosis, with rising rates of infections, partly due to increasing numbers of immunocompromised patients [3]. The significance of azole-resistance, first described in the 1990s [4, 5], raised in the last years [6] up to > 10% in some centers [7, 8]. Today, azole resistance in *A. fumigatus* is known to harbour a high priority for choosing the right treatment option.

Azole-resistance in *A. fumigatus* is acquired, and mainly occurs via tandem repeat mutations in the promoter region of the *cyp51A* gene in addition to amino acid substitution(s). *Cyp51A* encodes the enzymatic target of azole drugs, namely the sterol 14-alpha demethylase that plays a critical role in the ergosterol biosynthesis pathway of *A. fumigatus* [9]. The most frequently described azole-resistance underlying mutations of this gene are TR_34_/L98H and TR_46_/Y121F/T289A [10].

Early systemic antifungal treatment is crucial for patient outcome. The conventional treatment option for systemic infections with *A. fumigatus* such as invasive pulmonary aspergillosis (IPA) are triazoles, e.g. isavuconazole or voriconazole (VCZ). Liposomal amphotericin B (AMB) is another treatment option. While triazole treatment got challenging due to the upcoming of increased azole resistances, *Aspergillus* remained widely sensitive against treatment with liposomal AMB [11]. However, recently reported increasing AMB minimum inhibitory concentrations (MICs) of roughly 40% of Brazilian *A. fumigatus* isolates being resistant reveals more than ever the need for novel treatment options [12].

Several novel antifungal agents are on the rise. Fosmanogepix, the N-phosphonooxymethylene prodrug from the active moiety manogepix (MGX), previously showed good activity against a broad range of human pathogenic moulds and yeasts, among them *Aspergillus* [13]. MGX is a pyridine-isoxazole based antifungal, targeting enzymatic post-translational modification of proteins in fungi. Olorofim (OLO), former F901318, is the first drug of the new class of orotomides, targeting the pyrimidine biosynthesis. There is promising data on OLO activity against moulds, even though the FDA declined the first application of olorofim for treatment of rare mould infections in 2023 [14].

We here assessed the activity of AMB, itraconazole (ITR), OLO, MGX, and VCZ over time in a German ECMM Excellence Center against a total of 143 azole resistant *A. fumigatus* (ARAf) strains.

## Methods

### Isolates

The study did not include patients` details and did not result in additional constraints for patients. All data were anonymously analysed without patient consent owing to the retrospective nature of the study. All procedures and methods were carried out in accordance with approved guidelines. The isolates included in this study comprised a total of 143 clinical ARAf strains, collected between the years 2011 and 2022 (Table 1) at the ECMM Excellence Center, University Hospital Essen, Germany. Entry criterion for all isolates was growth on sabouraud dextrose agar containing 4 mg/L ITR [15] as well as minimum inhibitory concentrations of itraconazole and voriconazole > 1 mg/L proven by MIC gradient test. The species had been identified by characteristic micro- and macro-morphology as well as in parts by β-tubulin or ITS-sequencing as described previously [15]. All isolates were frozen at -80 °C until further use and are listed in supplementary Table 1.

**Table 1:** Number of strains collected per year. One isolate per patient

Year	N
2011	2
2012	18
2013	12
2014	7
2015	12
2016	12
2017	9
2018	10
2019	8
2020	17
2021	26
2022	10

### Broth Microdilution (BMD) Assay

BMD was carried out according to European Committee on Antimicrobial Susceptibility Testing (EUCAST) recommendations (E.DEF 9.4) for determination of broth dilution minimum inhibitory concentrations of antifungal agents for conidia forming moulds [16] for all 143 isolates. AMB (Sigma-Aldrich, St. Louis, MO, USA), MGX (APX001A; InvivoChem, Libertyville, IL, USA), ITR (Sigma-Aldrich), OLO (F901318; Hycultec, Beutelsbach, Germany), VCZ (Sigma-Aldrich) were diluted in solvent [dimethyl sulfoxide (DMSO)] and RPMI + MOPS [3-(N -morpholino)propanesulfonic acid] (2% glucose) for the preparation of stock solutions. The concentration ranges for the BMD assay are stated in Table 2. MICs or respectively minimum effective concentrations (MECs) were determined as cut-off values for each isolate after 48 h of incubation at 34 to 37 °C in ambient air. MICs were defined as the concentration yielding no discernible growth upon simple visual inspection [17]. MGX MECs were read using a confocal microscope as recommended by EUCAST [16, 18]. The MIC/MEC range, MIC_50_/MEC_50_, MIC_90_/MEC_90_, and geometric mean (GM) MIC/MEC were estimated. For calculation of GM, MIC values > 8 mg/L were defined as 16 mg/L.

**Table 2:** Test ranges (mg/L) in broth microdilution for the five included antifungal agents

AMB	ITR	MGX	OLO	VCZ
0.016–8	0.016–8	0.004–2	0.001–0.5	0.016–8

AMB, Amphotericin B; ITR, Itraconazole; MGX, Manogepix; OLO, Olorofim; VCZ, Voriconazole

As a quality control, *A. fumigatus* reference strain ATCC 204305 was included. For data interpretation, the breakpoint table version 10.0 provided by EUCAST was applied [19]. Breakpoints were used to interpret MICs obtained for AMB, ITR and VCZ, as no breakpoints are available for the other included agents.

### DNA Isolation

From Sabouraud dextrose agar inoculated with the isolate, three 5 mm^2^ agar blocks were punched out and lysed using MagNA Lyser (Roche, Basel, Switzerland). For total DNA extraction and purification, the Maxwell 16 instrument was used with the Maxwell 16 LEV Total RNA Purification Kit (Promega, Mannheim, Germany).

### Determination of Mutations in *cyp51A*

A commercially available multiplex real time PCR (AsperGenius® 1.0, Pathonostics, Maastricht, The Netherlands) was performed for all 143 isolates for detection of the most common mutations in *cyp51A:* TR_34_, L98H and Y121F/T289A.

In cases without TR_34_, L98H and Y121F/T289A mutations the amplification of the *cyp51A*-gene was done with three distinct primer pairs (Table 3). For those strains negative in the AsperGenius® PCR, *cyp51A* gene was sequenced as described elsewhere [20]. Amplification for these strains were followed by aligning of sequences in the FunResDB, a web-resourced database by the *Nationales Referenzzentrum für invasive Pilzinfektionen* (NRZMyk, Jena, Germany) for genotypic susceptibility testing of *A. fumigatus* [21]. The sequences were matched with the non-mutated *cyp51A* sequence.

**Table 3:** Sequences of three primer pairs used for PCR. Primers were used in concentration of 50 µM, the final concentration was 12.5 µM

Primer	Forward primer sequence	Reverse primer sequence
CYP1	5’-CACCCTCCCTGTGTCTCCT-3’	5’-AGCCTTGAAAGTTCGGTGAA-3’
CYP2	5’-CATGTGCCACTTATTGAGAAGG-3’	5’-CCTTGCGCATGATAGAGTGA-3’
CYP3	5’-TTCCTCCGCTCCAGTACAAG-3’	5’-CCTTTGAAGTCCTCGATGGT-3’

## Results

The here tested antifungal agents showed different activity against the ARAf strains. The obtained MIC_50_, MIC_90_ values as well as the GM and range of MICs and MEC, respectively are listed for each tested antifungal drug in Table 4. Overall, the lowest MIC/MECs have been shown for MGX followed by OLO against the vast majority of included strains. Considering the clinical breakpoints provided by EUCAST, 1.4, 92.3 and 97.9% of the strains were resistant towards AMB, ITR, and VCZ, respectively.

**Table 4:** Minimum inhibitory/effective concentrations (mg/L) of ARAf (N = 143) against five different antifungal agents

	AMB	ITR	MGX*	OLO	VCZ
GM^#^	0.540	10.664	0.018	0.029	4.747
Range	0.125–2	0.5–> 8	0.08–0.06	0.008–0.125	0.25–> 8
MIC_50_/MEC_50_	0.5	> 8	0.016	0.03	4
MIC_90_/MEC_90_	1	> 8	0.03	0.06	> 8

AMB, Amphotericin B; ITR, Itraconazole; GM, Geometric mean; MGX, Manogepix; MEC_50_, Minimum effective concentration for at least 50% of strains; MEC_90_, Minimum effective concentration for at least 90% of strains; MIC_50_, Minimum inhibitory concentration for at least 50% of strains; MIC_90_, Minimum inhibitory concentration for at least 90% of strains; OLO, Olorofim; VCZ, Voriconazole. *MEC was determined for MGX. ^#^When no detectable antifungal activity was observed at 8 mg/L a value of 16 mg/L was used to calculate the GM

**Fig. 1:**
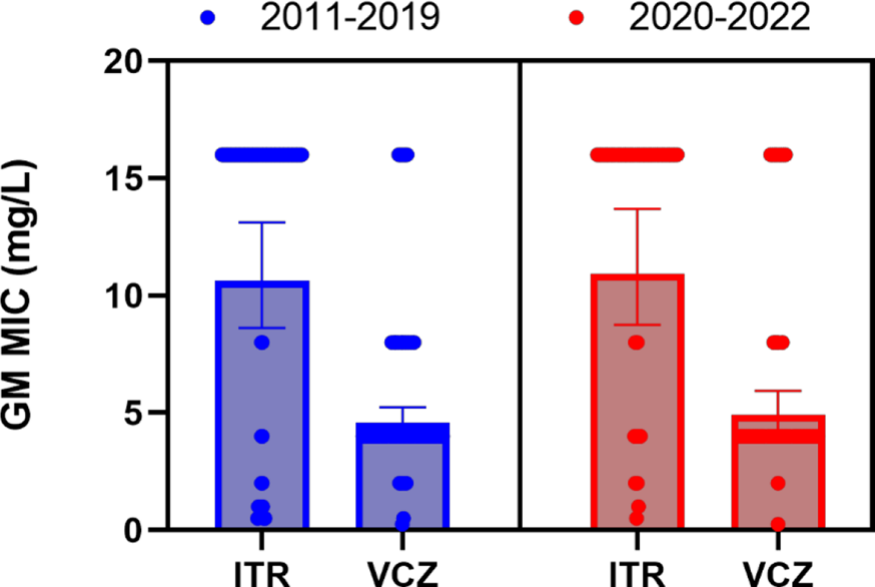
Geometric mean (GM) with 95% confidence interval of itraconazole (ITR) and voriconazole (VCZ) in mg/L against ARAf collected in the years 2011 to 2019 (blue, N = 90)) and 2020–2022 (red, N = 53). When no detectable antifungal activity was observed at 8 mg/L a value of 16 mg/L was used to calculate the GM

A comparison of triazole MICs between the period from 2011 and 2019 compared with the MICs of isolates collected between 2020 and 2022 revealed no significant differences (*p* = 0.534 for itraconazole and 0.148 for voriconazole, paired t test), with slightly increased GM value for both triazoles over time (GM itraconazole 2011–2019 = 10.6 mg/L; 2020–2022 = 10.9 mg/L and GM voriconazole 2011–2019 = 4.7 mg/L; 2020–2022 = 4.9 mg/L; Fig. 1).

Molecular investigation of the strains revealed that the vast majority (86%) of ARAf strains have an underlying TR_34_/L98H mutation, followed by TR_46_/Y121F/T289A with 7% whereas 7% of strains harboured other *cyp51A*-associated point mutations. The identified non TR_34_/L98H and TR_46_/Y121F/T289A mutations are listed in Table 5.

**Table 5:** Mutations detected in cyp51A sequencing for ARAf strains (N = 10) without a TR_34_/L98H/TR_46_/Y121F/T289A mutation

ID		TR in DNA promoter	Mutations
8	1964	NO	G448S
10	1966	NO	F46Y, Y158D, L159R, M172V, I360F, H361L, L336F, E427K, K515R, *516R
15	1972	No	F46Y, D157P, S168K, H147P, L150P, M172V, N248T, D255E, M368L, R369N, K370L, K372I, E427K
20	1979	NO	M4T, W6G, L7I, L159P, R160G, S168Q, S146L, H147P, L150P, M368R, V371E, L336F
87	2344	NO	K153N, E154D, L156F, D157C, Y158N, H285Y, S362I, V371E, L336F, L347R, P348S
106	2488	NO	L7D, K153N, E154P, V155L, D157G, S168*, E154R, P163T, P216H, I364H, V371G, L336F, K515R, *516R
167	2795	NO	N164T
183	M328	NO	M1K, L7I, G54W, V155E, Y158L, Q166K, S168*, D157S, L159Q, R160A, D161N, S162C, P163T, T357P, H361Y, S362V, V371E, L336F, K346Q, K511I, K515R, *516R
189	M414	NO	L7V, V13I, R160C, S168*, Y158S, S162A, R369G, L336F, K511I, K515R, *516R
208	M923	NO	V155A, V155S, R369A, L336F, P348S, T513N

TR, Tandem repeat

**Fig. 2:**
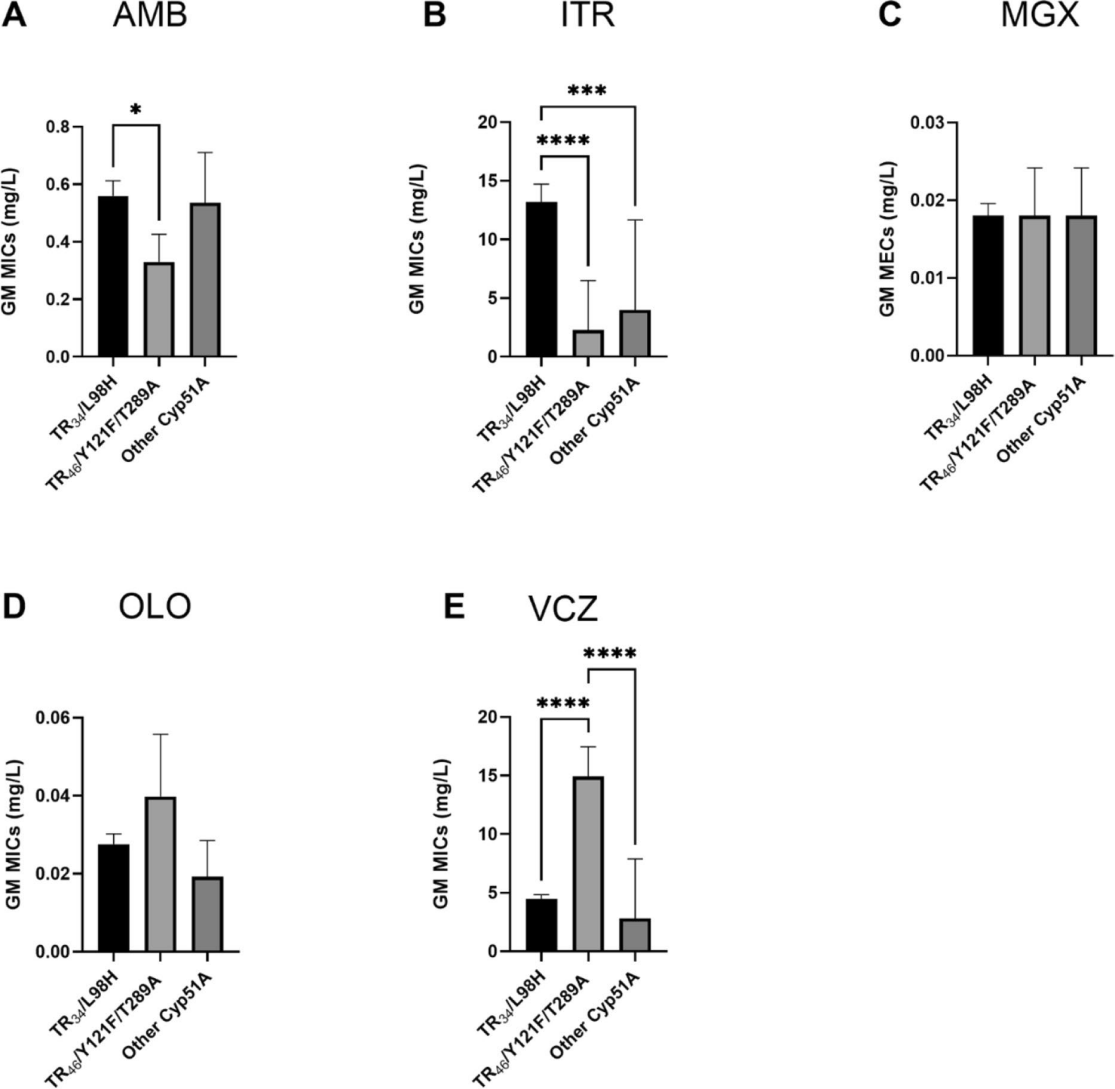
Geometric Mean (GM) with 95% Confidence interval of **a** minimum inhibition concentration (MIC) of amphotericin B (AMB), **b** MIC of itraconazole (ITR), **c** MEC of manogepix (MGX), **d** MIC of olorofim (OLO) and **e** MIC of voriconazole (VCZ) towards A. fumigatus isolates with distinct underlying mutations: TR_34_/L98H (N = 123), TR_46_/Y121F/T289A (N = 10) and other unknown mutations (N = 10). Statistical test: ordinary one-way ANOVA. Significance was assumed when *p* < 0.05. *: *P* < 0.05; **** *p* < 0.0001. When no detectable antifungal activity was observed at 8 mg/L a value of 16 mg/L was used to calculate the GM

Concerning the azole resistance underlying mutations, the activity of AMB, ITR and VCZ did significantly differ (Fig. 2A–F). While VCZ did exhibit highest MICs against strains with mutations in TR_46_/Y121F/T289A, ITR did show significantly higher MICs towards the isolates with a TR_34_/L98H mutation. Even though, nearly all (97.9%) of the strains included in this analysis showed a VCZ resistance phenotype and 92.3% exhibited resistance to ITR according to EUCAST breakpoints. While four isolates (40%) with a TR_46_/Y121F/T289A mutation were susceptible towards ITR (MIC of 0.5 mg/L), only 5.7% of the TR_34_/L98H harbouring strains were susceptible against ITR. VCZ showed MICs ≥ 8 mg/L for all of the strains with TR_46_ mutation whereas the GM of MICs against the strains harbouring a TR_34_/L98H mutation was 4.5 mg/L. Only two (20%) strains (ID 106 and 183) with other mutations were susceptible towards VCZ (GM = 0.25 mg/L). For these strains, concordant mutations were S168*, P163T, L336F, K515R and *516R. For the novel antifungal agents MGX and OLO, no significant difference in activity against strains with distinct mutations has been noticed (Fig. 2D–E).

**Fig. 3:**
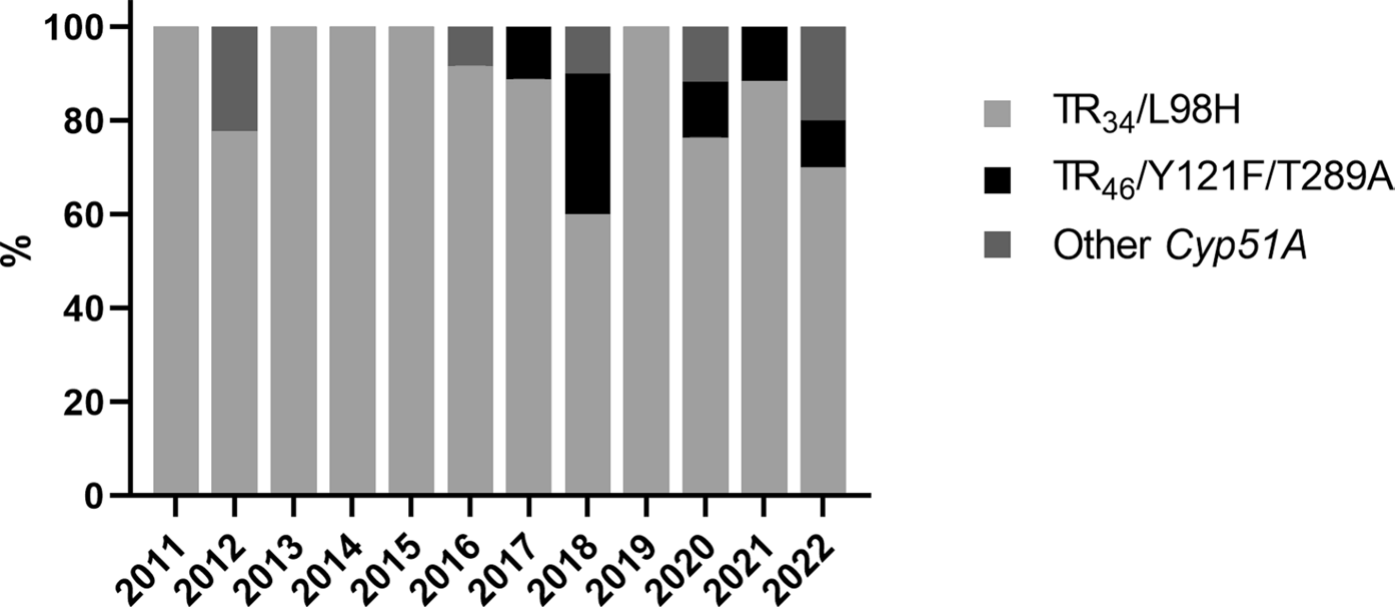
Prevalence of azole resistance underlying mutation in the included *A. fumigatus* strains over time

Over time, the occurring azole resistance underlying mutations in the collected ARAf strains varied (Fig. 3). While nearly all strains from clinical specimen before 2016 were TR_34_/L98H strains, the portion of strains with TR_46_/Y121F/T289A and other *cyp51A* mutated strains increased after that.

## Discussion

In this study, a total of 143 ARAf strains from a German ECMM excellence centre were analysed on their susceptibility towards the commonly used triazoles ITR and VCZ as well as AMB and the novel antifungal agents OLO and MGX. The findings for antifungal activity in BMD was overall good for all two tested novel agents, without significant differences in susceptibility of ARAf strains with distinct underlying mutations. The activity of the two novel drugs were additionally higher compared to those of the azoles and AMB on an mg/L basis.

In the Netherlands, the rates of azole resistance showed a declining trend over the past years, with varying frequency in individual centers, with the most common found mutation in TR_34_ [22, 23], going along with the here described results. Sequencing of isolates revealed most isolates harbouring the TR_34_/L98H mutation in the *cyp51A* gene. This is a 34-base pair tandem repeat and additionally a change of ‘L’ to ‘H’ at position 98. This mutation was rapidly emerging, especially in the Netherlands, but now has been increasingly identified in other, also non-European countries [24, 25]. Whereas isolates from The Netherlands, Taiwan, Denmark, Brazil and China were genetically similar, TR_34_/L98H strains from e.g. the Middle East are genotypically distinct from the European isolates, indicating no simple geographical spread [26]. However, other more recently published studies indicate high levels of similarities between isolates from the Netherlands, Taiwan, Denmark, Brazil, and China [25].

Risk factor for acquisition of infections with ARAf are e.g. presence and treatment of chronic *Aspergillus* infections, and a linked high fungal burden, and prolonged azole exposure [27]. Infections with ARAf isolates have been described to occur by two different routes, the development in situ in the lungs or by acquisition from the environment, potentially in parts driven by triazole use in agriculture [27]. The presence of TR_34_ and TR_46_ is indicating an acquisition from the environment, rather than the in-host resistance development [22]. Although this could not be proven in a recent publication [28]. In a study from 2021, ten isolates (2.5%) were non-WT to ITR and six of these were additionally non-WT to VCZ. Accordingly to our findings, Pfaller et al. found the most common substitution in *Cyp51A* being TR_34_/L98H [29]. A TR_53_ mutated strain could not been found in the here included strains. For the two voriconazole susceptible strains with other mutations current literature did not provide specific information about the mutations found in relation to voriconazole susceptibility.

*A. fumigatus* has been identified as the most common fungal pathogen occurring in critically ill COVID-19 patients and the prevalence of fungal drug resistance in patients suffering COVID-19 is generally high [30–32]. Here, the comparison of the MICs of both azoles against isolated ARAfs from clinical specimens in the pre-COVID-19 period from 2011 to 2019 and the post-COVID-19 era between 2020 and 2022 revealed no significant differences in GM MICs. However, a trend of increasing MICs of both azoles could be detected. In contrast, the Dutch surveillance network reported a decreasing trend in the mean VCZ MIC values amongst *A. fumigatus* isolates harbouring the TR_34_/L98H mutation from 8 mg/L in 2013 to 2 mg/L in 2018 and the resistance rates towards VCZ declined around 34% in this time [23]. Here, comparing the GM MICs of VCZ against pre- and post-COVID isolated ARAFs with a TR_34_/L98H mutation, an increasing trend without statistical significance could be detected.

Notably, the here obtained MIC data of both triazoles against *A. fumigatus* revealed significantly higher MICs of VCZ against strains with a TR_46_/Y121F/T289A in *cyp51A* compared to TR_34_/L98H_,_ whereas in case of ITR, higher MICs were found against strains with a TR_34_/L98H mutation. The differences in susceptibility phenotypes with regard to the underlying mutations and its specific combinations was beforehand described in literature [33–36]. As a limitation resulting from the selected PCR for detection of mutations, our study did not search for additional mutations, which are simultaneously present together with TR_46_/Y121F/T289A and TR_34_/L98H, e.g. G448S. Although isolates with several azole resistance underlying mutations are mainly found in the environment [37] than within patients’ settings [38], our diagnostic approach might have missed other mutations. Another limitation is that no total numbers of ARAf and wildtype *A. fumigatus* isolates from the same period were given to build proportions in order to compare our findings with other countries.

MGX has previously been demonstrated to be active against a broad range of human fungal pathogens, including *Candida* spp., difficult-to-treat rare moulds as *Scedosporium* spp. and *Aspergillus* spp. [39–43]. In a study on 397 *A. fumigatus* isolates analysed in CLSI broth microdilution method, MEC_50_ of 0.015 mg/L, MEC_90_ of 0.03 mg/L was determined for MGX [29]. The determined MEC_50_ and MEC_90_ values are corresponding to the data we collected. Additionally, MGX susceptibility did not differ between wild type *A. fumigatus* and ARAf strains as described by Pfaller et al*.* and has been described to have similar activity compared to echinocandins against *A. fumigatus*. In this study on 379 *A. fumigatus* strains, a wild-type upper limit (WT-UL) MECs of ≤ 0.06 mg/L was defined for MGX [29]. Using this as an epidemiological cut-off value (ECOFF) equivalent for the here generated data, 2.1% (N = 3) of the ARAf strains were non-wild-type towards MGX. In contrast, 100% of the *A. fumigatus* strains assessed by Pfaller et al. were MGX wild-type [29].

OLO has been demonstrated to exhibit activity against various moulds, including *A. fumigatus* wild type and ARAf [44, 45]. In a study from 2020 on 25 ARAf isolates, a MIC range from ≤ 0.008–0.032 mg/L was detected which is lower than the here defined highest MIC of 0.125 mg/L [44]. Other studies found MICs between 0.016 and 0.25 mg/L [45–47]. No difference in MIC distribution was detected between azole-susceptible and-resistant isolates [45]. An assessment of 975 clinical *A. fumigatus* isolates revealed no intrinsic OLO resistance. In contrast, high numbers of conidia and olorofim exposure could force acquired OLO resistant phenotypes (MICs > 8 mg/L) under laboratory conditions. This frequency of acquired resistance was shown to be lower than that for ITR but higher than for VCZ. The authors were furthermore capable to identify several amino acid substitutions in the *PyrE* gene with high rates at G119V [48]. In a study on OLO antifungal activity, a WT-UL value of 0.03 mg/L was determined for *A. fumigatus *sensu lato strains [49]. In another study on OLO activity against 1032 *A. fumigatus* isolates, a WT-UL_97.5_ of ≤ 0.125 mg/L was defined [45]. Taking this WT-UL value as an ECOFF equivalent, here one strain (0.7%) showed non wild-type olorofim MICs.

In conclusion, ARAfs are still a threat for patients and face their treating physicians with therapeutic challenges. We found almost stable MICs for ITR and VCZ over twelve years in ARAf isolates from West Germany while occurring azole resistance underlying mutations varied with an increase in the proportion of TR46/Y121F/T289A and other Cyp51A mutations in ARAf iafter 2016. The novel antifungals showed excellent in vitro activity against all type of mutations.
